# Performance and usability of machine learning for screening in systematic reviews: a comparative evaluation of three tools

**DOI:** 10.1186/s13643-019-1222-2

**Published:** 2019-11-15

**Authors:** Allison Gates, Samantha Guitard, Jennifer Pillay, Sarah A. Elliott, Michele P. Dyson, Amanda S. Newton, Lisa Hartling

**Affiliations:** 1 Department of Pediatrics, Alberta Research Centre for Health Evidence and the University of Alberta Evidence-based Practice Center, University of Alberta, 11405 87 Ave NW, Edmonton, Alberta T6G 1C9 Canada; 2 Department of Pediatrics, University of Alberta Evidence-based Practice Center, University of Alberta, 11405 87 Ave NW, Edmonton, Alberta T6G 1C9 Canada

**Keywords:** Systematic reviews, Machine learning, Automation, Usability, User experience

## Abstract

**Background:**

We explored the performance of three machine learning tools designed to facilitate title and abstract screening in systematic reviews (SRs) when used to (a) eliminate irrelevant records (automated simulation) and (b) complement the work of a single reviewer (semi-automated simulation). We evaluated user experiences for each tool.

**Methods:**

We subjected three SRs to two retrospective screening simulations. In each tool (Abstrackr, DistillerSR, RobotAnalyst), we screened a 200-record training set and downloaded the predicted relevance of the remaining records. We calculated the proportion missed and workload and time savings compared to dual independent screening. To test user experiences, eight research staff tried each tool and completed a survey.

**Results:**

Using Abstrackr, DistillerSR, and RobotAnalyst, respectively, the median (range) proportion missed was 5 (0 to 28) percent, 97 (96 to 100) percent, and 70 (23 to 100) percent for the automated simulation and 1 (0 to 2) percent, 2 (0 to 7) percent, and 2 (0 to 4) percent for the semi-automated simulation. The median (range) workload savings was 90 (82 to 93) percent, 99 (98 to 99) percent, and 85 (85 to 88) percent for the automated simulation and 40 (32 to 43) percent, 49 (48 to 49) percent, and 35 (34 to 38) percent for the semi-automated simulation. The median (range) time savings was 154 (91 to 183), 185 (95 to 201), and 157 (86 to 172) hours for the automated simulation and 61 (42 to 82), 92 (46 to 100), and 64 (37 to 71) hours for the semi-automated simulation. Abstrackr identified 33–90% of records missed by a single reviewer. RobotAnalyst performed less well and DistillerSR provided no relative advantage. User experiences depended on user friendliness, qualities of the user interface, features and functions, trustworthiness, ease and speed of obtaining predictions, and practicality of the export file(s).

**Conclusions:**

The workload savings afforded in the automated simulation came with increased risk of missing relevant records. Supplementing a single reviewer’s decisions with relevance predictions (semi-automated simulation) sometimes reduced the proportion missed, but performance varied by tool and SR. Designing tools based on reviewers’ self-identified preferences may improve their compatibility with present workflows.

**Systematic review registration:**

Not applicable.

## Background

There is growing recognition that expedited systematic review (SR) processes are needed to keep pace with the rapid publication of primary studies [1]. Title and abstract screening is a review step that may be particularly amenable to automation or semi-automation [2–4]. There is increasing interest in ways that reviewers can leverage machine learning (ML) tools to expedite screening while maintaining SR validity [5]. One way that ML tools expedite screening is by predicting the relevance of remaining records after reviewers screen a “training set.” What remains unclear is how and when reviewers may reliably leverage these predictions to semi-automate screening. Reviewers would benefit from understanding the relative reliability, usability, learnability, and costs of available tools.

A review of published studies on applications of ML tools for screening found that they could be used safely to prioritize relevant records and cautiously to replace the work of one of two human reviewers [6]. Despite their promise, the adoption of ML tools among reviewers has been slow [6–8]. O’Connor et al. summarized potential barriers to adopting ML tools among reviewers. Concerns included distrust in ML approaches by reviewers and end users, set-up challenges and incompatibility with standard workflows, doubts as to whether ML tools can reliably perform SR tasks, and poor awareness of available tools [9].

In light of known barriers to ML tool adoption [9–12], we investigated the relative advantages and risks of using ML tools to automate or semi-automate title and abstract screening. For three SRs, we compared how three ML tools performed when used in the context of (a) single reviewer screening to eliminate irrelevant records and (b) dual independent screening to complement the work of one of the human reviewers. We also aimed to compare user experiences across the tools.

## Methods

### Conduct

We followed an a priori protocol, available upon request.

### Machine learning tools

We investigated Abstrackr (http://abstrackr.cebm.brown.edu), DistillerSR (the ML tool being DistillerAI) (http://www.evidencepartners.com), and RobotAnalyst (http://www.nactem.ac.uk/robotanalyst/). From a user’s perspective, the three tools function similarly. After uploading citations to the user interface, titles and abstracts appear on-screen and reviewers are prompted to label each as relevant or irrelevant. The ML algorithms use reviewers’ relevance labels and other data (e.g., relevance terms tagged by reviewers, text mining for MeSH terms and keywords) to predict which of the remaining records are relevant.

Although many ML tools exist [13], we chose Abstrackr, DistillerSR, and RobotAnalyst because their development is well-documented [14–16], and at least for Abstrackr and RobotAnalyst, real-world performance has been evaluated [17–19]. We also chose the tools for practical reasons. All three allow the user to download the relevance predictions after screening a training set. Both Abstrackr and RobotAnalyst are freely available, and although DistillerSR is a pay-for-use software, our center maintains a user account.

### Performance testing

#### Screening procedure

We selected a convenient sample of three SRs of healthcare interventions completed or underway at our center (Table 1). For each SR, we uploaded all records to each tool via RIS (Research Information Systems) files. We set up the SRs for single-reviewer screening with the records presented in random order. Although we had intended to use the “most likely to be relevant” prioritization, we were not successful in applying this setting in all tools (due to server errors or glitches in Abstrackr and RobotAnalyst).

**Table 1:** Population, intervention, comparator, outcome, and study design (PICOS) criteria for the systematic reviews

Criteria	Antipsychotics [20]	Bronchiolitis	Visual Acuity [21]
Population	Children and young adults aged ≤ 24 years experiencing a psychiatric disorder or behavioral issues outside the context of a disorder	Infants and young children aged < 24 months experiencing their first episode of wheeze or diagnosed with bronchiolitis or RSV	Community-dwelling adults aged ≥ 65 years with unrecognized impaired visual acuity or vision-related functional limitations
Intervention	Any Food and Drug Administration-approved first- or second-generation antipsychotic	Any bronchodilator, any corticosteroid, hypertonic saline, oxygen therapy, antibiotics, heliox	Vision screening tests (alone or within multicomponent screening/assessment) performed by primary healthcare professionals
Comparators	Placebo, no treatment, any other antipsychotic, the same antipsychotic in a different dose	Placebo, usual care, no treatment, normal saline, or another intervention of interest	No screening, delayed screening, attention control, screening involving all components of intervention except vision component, usual care
Outcomes	Intermediate and effectiveness outcomes, adverse effects and major adverse effects, adverse effects limiting treatment, specific adverse events, persistence and reversibility of adverse effects	Outpatient admissions, inpatient length of stay, change in clinical score, oxygen saturation, respiratory rate, heart rate, pulmonary function, adverse events, escalation of care, length of illness, duration of oxygen therapy	Benefits (e.g., mortality, adverse consequences of poor vision), harms (e.g., serious adverse events), implementation factors (e.g., uptake of referrals)
Study designs	RCTs and nRCTs, controlled cohort studies, controlled before-after studies	RCTs	RCTs, controlled experimental and observational studies

*nRCT* non-randomized controlled trial, *RCT* randomized controlled trial, *RSV* respiratory syncytial virus

When using ML tools for screening, inaccurate labels in the training set will result in unreliable predictions. Thus, for a training set of 200 records, we retrospectively replicated the senior reviewer’s (the reviewer with the most content expertise or SR experience) screening decisions in each tool. In a previous evaluation [18], we found 200 records to be sufficient to develop predictions. The developers of DistillerAI recommend a minimum training set size of 40 excluded and 10 included records and a maximum size of 300 records [22]. Because the records appeared in random order, the training set differed across the tools for each review. Although this could affect the predictions, in a previous evaluation, we found little difference in Abstrackr’s predictions over three independent trials [15].

At our center, any record marked as “include” or “unsure” by either reviewer during title and abstract screening is eligible for scrutiny by full text. For this reason, our screening files included one of two screening decisions for each record: include/unsure or exclude. Because we were unable to retrospectively ascertain whether the decision for individual records was “include” or “unsure,” we entered all “include/unsure” decisions as “relevant.”

After screening the training sets, we downloaded the relevance predictions for the remaining records in each tool. In DistillerSR and RobotAnalyst, these were available immediately. In Abstrackr, they were typically available the following day. When the predictions did not become available within 48 h, we continued to screen in batches of 100 records until they did. The format of the predictions varied by tool. Abstrackr produced “hard screening predictions” (true, i.e., include or false, i.e., exclude) and relevance probabilities for each remaining record. We used the hard screening predictions rather than applying custom thresholds based on the probabilities. Both DistillerSR and RobotAnalyst provided binary predictions (include or exclude) for all remaining records. Although customization was possible in DistillerSR, we used the “simple review” function to automatically classify the remaining records.

#### Retrospective simulations

Based on existing reviews [2, 6, 11], we postulated that the ML tools’ relevance predictions could be leveraged to (a) automatically exclude irrelevant records or (b) complement the work of one of the human reviewers. We devised two retrospective screening simulations to test our hypothesis. In the first approach (automated simulation, the automatic exclusion of records), after screening the training set, the senior reviewer downloaded the predictions and excluded all records predicted to be irrelevant. To reduce the full-text screening workload, the reviewer continued to screen the records predicted to be relevant. Of these, the records that the reviewer agreed were relevant moved forward to full-text screening. In the second approach (semi-automated simulation, complementing the work of one human reviewer), we aimed to determine whether the predictions could be leveraged to improve upon the work of a single reviewer (as naturally, a single reviewer can be expected to erroneously exclude relevant records) [23]. In this simulation, the senior reviewer followed the same approach as in the automated simulation, and the second reviewer screened all of the records as per usual. Any record marked as relevant by the second reviewer or the senior reviewer/tool’s predictions moved forward to full-text screening.

To test the performance of each approach, we created a workbook in Excel (v. 2016, Microsoft Corporation, Redmond, Washington) for each SR. The workbooks included a row for each record and a column for each of the record identification number, the title and abstract screening decisions for the senior and second reviewers, the full-text consensus decisions, and the relevance predictions from each tool. We then determined the title and abstract decisions that would have resulted from each simulation. As per standard practice at our center, we considered any record marked as “include” by either of the reviewers to be relevant for scrutiny by full text.

### User experience testing

In February 2019, we approached a convenient sample of 11 research staff at our center to participate in the user experience testing. These staff were experienced in producing SRs (e.g., research assistants, project coordinators, research associates), but had no or very little experience with ML tools for screening. We allowed invited participants 1 month to undertake the study, which entailed completing a screening exercise in each tool and a user experience survey. Participation was voluntary and completion of the survey implied consent. We received ethical approval for the user experience testing from the University of Alberta Research Ethics Board (Pro00087862).

We designed a screening exercise that aligned with practices at our center (Additional file 1). The aim of the exercise was to guide participants through the steps involved in setting up a SR, uploading a set of records, screening a training set, and downloading the predictions in each tool. We provided minimal guidance only instructing participants to use the “Help” function in each tool if needed.

For the screening exercise, we selected a SR with relatively straightforward eligibility criteria that was underway at our center (PROSPERO #CRD42017077622). We wanted participants to focus on their experience in each tool and did not want complex screening criteria to be a distraction. To reduce the risk of response bias, we used the random numbers generator in Excel to randomize the order in which each participant tested the three tools.

The survey (Additional file 2), hosted in REDCap (Research Electronic Data Capture) [24], asked participants to complete the System Usability Scale (SUS) [25] for each tool. The SUS is a 10-item questionnaire that assesses subjective usability using a Likert-like scale [25]. The survey also asked participants to elaborate on their experiences with each tool, rank the tools in order of preference, and describe the features that supported or detracted from their usability.

We made minor changes to the screening exercise (reduced the suggested number of citations to screen to minimize participant burden) and survey (edited for typos) following pilot testing by two researchers at our center. Because the changes were minimal, we retained the data from the two researchers for analysis, with permission.

### Analysis

#### Performance

We exported the simulation data from Excel to SPSS Statistics (v. 25, IBM Corporation, Armonk, New York) for analysis. We used data from 2 × 2 cross-tabulations to calculate standard [6] performance metrics for each simulation, as follows:

- *Proportion of records missed* (*i.e*., *error*): of the studies included in the final report, the proportion that would have been excluded during title and abstract screening.
  We made informal comparisons of the proportion missed for each simulation and tool to single reviewer screening to estimate the acceptability of its performance.
- *Workload savings* (*i*.*e*., *absolute screening reduction*): of the records that need to be screened at the title and abstract stage, the proportion that would not need to be screened manually.
- *Estimated time savings*: the time saved by not screening records manually. We assumed a screening rate of 0.5 min per record [26] and an 8-h work day.

Additional file 3 shows sample calculations for the Antipsychotics SR using Abstrackr’s predictions.

#### User experiences

We exported the quantitative survey data from REDCap to Excel for analysis and the qualitative survey data to Word (v. 2016, Microsoft Corporation, Redmond, Washington). For each participant, we calculated the overall usability score for each tool as recommended by Brooke [25]. We calculated the median and interquartile range of scores for each tool and categorized their usability as recommended by Bangor et al. [27]: not acceptable (0 to 50), marginal (50 to 70), and acceptable (70 to 100). For the ranking of tools by preference, we calculated counts and percentages.

We analyzed the qualitative data following standard, systematic approaches to thematic analysis [28]. One researcher (AG) read the text and applied one or more codes to each line. Next, the researcher identified the most significant and frequent codes, combined similar codes, and developed memos for each theme. To reduce the risk of interpretive bias, a second researcher external to the study team reviewed the analysis for differences in interpretation. All disagreements were resolved via discussion.

## Results

### Performance

Table 2 shows the screening characteristics for each SR. The screening workload ranged from 5861 to 12,156 records. Across SRs, 2–10% of records were retained for scrutiny by full text. Across SRs, ≤ 2% of all records were included in the final reports. The Visual Acuity review was unique in that only one record from the 11,229 screened was included in the final report. The final reports for the Antipsychotics and Bronchiolitis reviews included 127/12156 and 137/5861 records, respectively.

**Table 2:** Characteristics of the reviews and screening predictions for each tool

Characteristic	Antipsychotics, *N* records (%)	Bronchiolitis, *N* records (%)	Visual Acuity, *N* records (%)
Screening workload^a^	12,156	5861	11,229
Included by title/abstract^b^	1178 (10)	518 (9)	224 (2)
Included in the review^b^	127 (1)	137 (2)	1 (< 1)
Includes/excludes in training set	Abstrackr, 15/185	Abstrackr, 12/188	Abstrackr^c^, 4/296
	DistillerSR, 14/186	DistillerSR, 14/186	DistillerSR, 2/198
	RobotAnalyst, 20/180	RobotAnalyst, 15/185	RobotAnalyst, 3/197
Screened by tool^d^	11,956 (98)	5661 (97)	11,029 (98)
Predicted relevant by Abstrackr	2117 (18)	656 (12)	3639 (33)
Predicted relevant by DistillerSR	7 (< 1)	83 (1)	0 (0)
Predicted relevant by RobotAnalyst	3488 (29)	1082 (19)	3221 (29)

^a^Total number of records retrieved via the electronic searches. Each record was screened by two reviewers

^b^Included following the initial screening by two independent reviewers (retrospective)

^c^All training sets were 200 records, with the exception of the Visual Acuity review which required a 300-record training set in Abstrackr before predictions were produced

^d^After a 200-record training set

Predictions were available after screening 200 records for all SRs in all tools with the exception of Visual Acuity in Abstrackr. As planned, we screened an additional 100 records, and the predictions became available. For two of the SRs, RobotAnalyst did not upload the full list of records from the RIS file. Because all of our troubleshooting attempts (at least six attempts and contact with the developers) failed, we assumed that the additional 170 records for Bronchiolitis and 183 records for Visual Acuity would need to be screened manually. We thus used the human reviewers’ original decisions (include or exclude) when applying the simulations.

In Abstrackr, DistillerSR, and RobotAnalyst, the training sets included a median (range) of 12 (4, 15), 14 (2, 14), and 15 (3, 20) includes respectively, with the balance being excludes. After screening the training sets, Abstrackr, DistillerSR, and RobotAnalyst predicted that a median (range) 18 (12, 33)%, 0.1 (0, 1)%, and 29 (20, 29)% of the remaining records were relevant, respectively. Cross-tabulations showing records included in the final report relative to those deemed relevant via each simulation are in Additional file 4.

### Automated simulation

#### Proportion missed

**Fig. 1:**
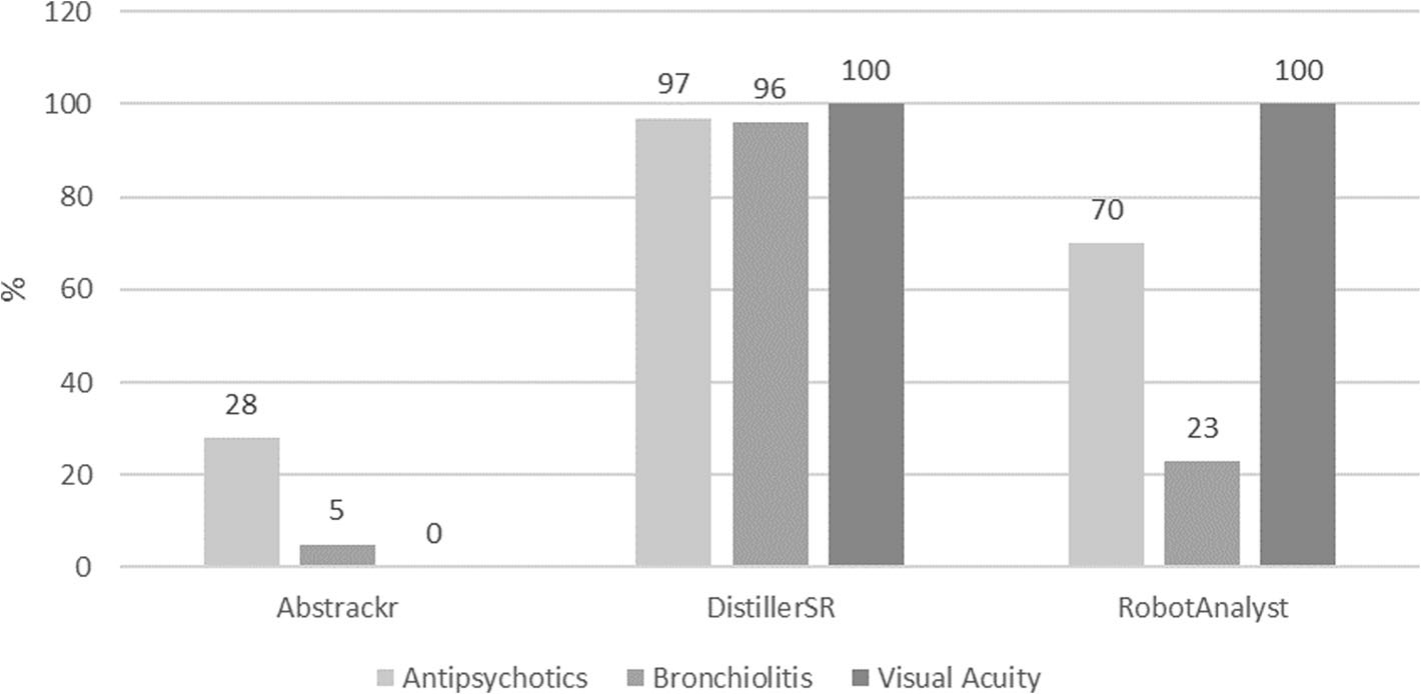
Proportion missed (percent) by tool and systematic review, automated simulation

Records “missed” are those that would not have moved forward to full-text screening, but were included in the final reports. The median (range) proportion missed was 5 (0, 28)%, 97 (96, 100)%, and 70 (23, 100)% using Abstrackr, DistillerSR, and RobotAnalyst, respectively (Fig. 1).

#### Workload savings

**Fig. 2:**
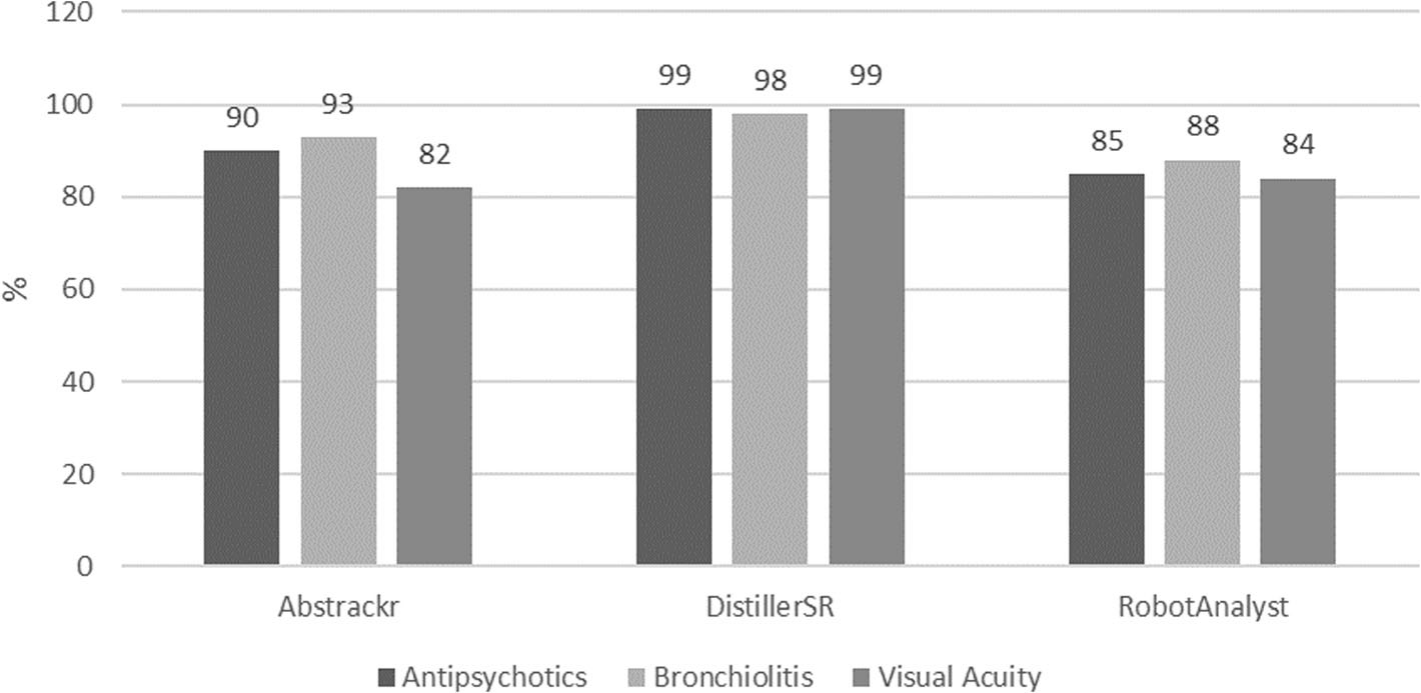
Workload savings (percent) by tool and systematic review, automated simulation

The median (range) workload savings was 90 (82, 93)%, 99 (98, 99)%, 85 (84, 88)% for Abstrackr, DistillerSR, and RobotAnalyst, respectively (Fig. 2).

#### Estimated time savings

**Fig. 3:**
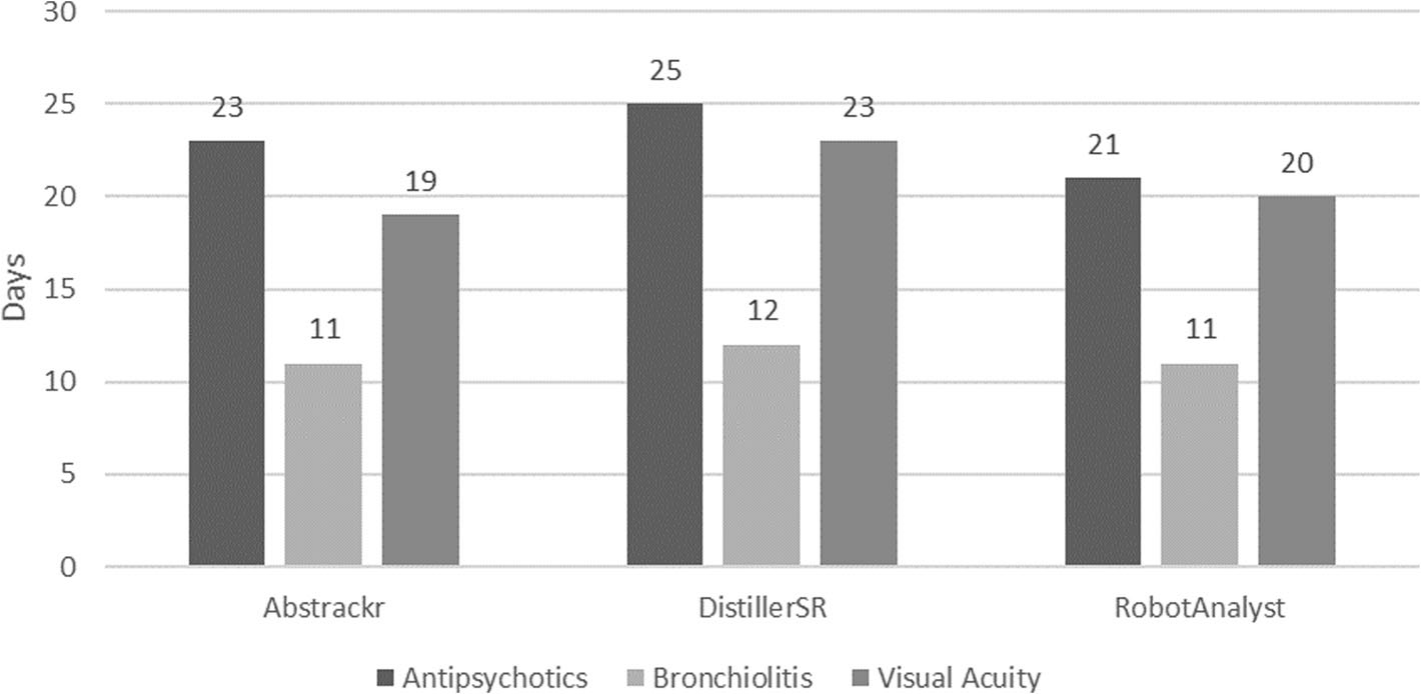
Estimated time savings (days) by tool and systematic review, automated simulation

The median (range) time savings was 154 (91, 183), 185 (95, 201), and 157 (86, 172) hours for Abstrackr, DistillerSR, and RobotAnalyst, respectively (i.e., a respective 19 (11, 23), 23 (12, 25), and 20 (11, 21) days) (Fig. 3).

### Semi-automated simulation

#### Proportion missed

**Fig. 4:**
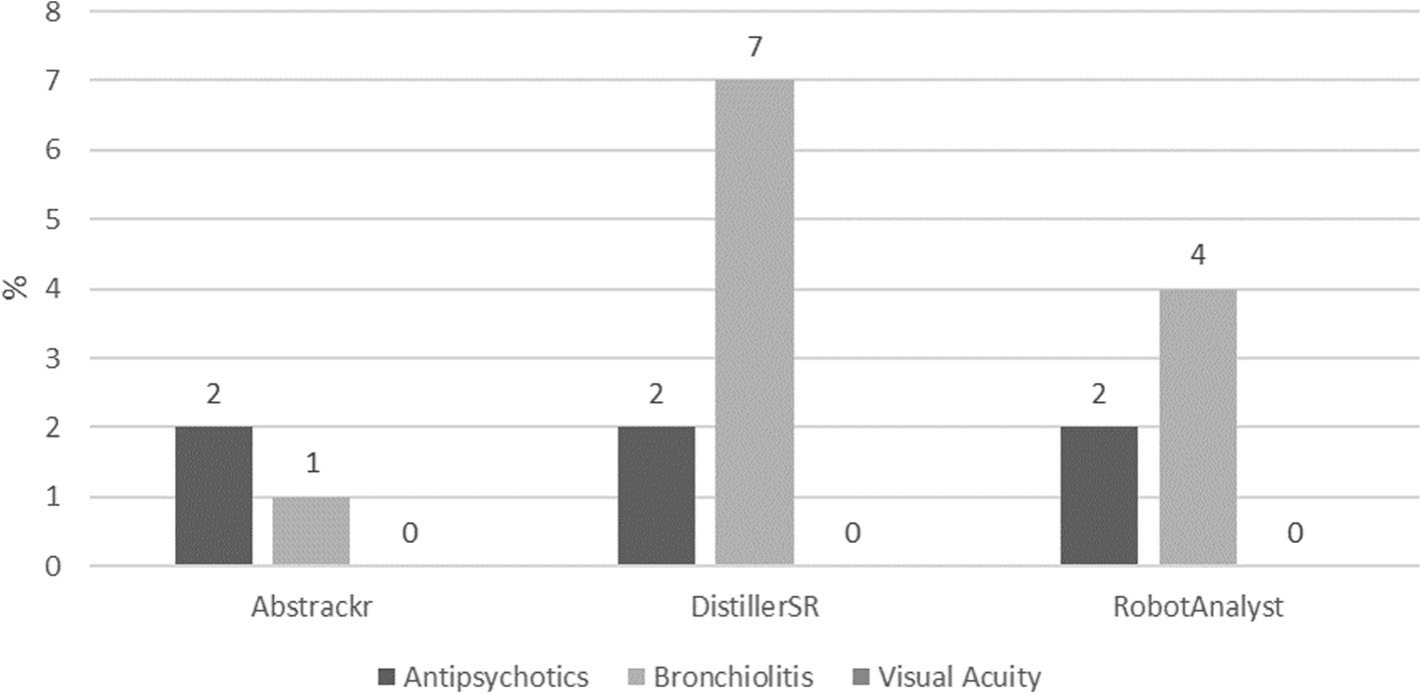
Proportion missed (percent) by tool and systematic review, semi-automated simulation

The median (range) proportion missed was 1 (0, 2)%, 2 (0, 7)%, and 2 (0, 4)% for Abstrackr, DistillerSR, and RobotAnalyst, respectively (Fig. 4). Important to the performance of the semi-automated simulation is the contribution of each tool’s predictions to the overall screening accuracy. Had the second reviewer screened the records for Antipsychotics, Bronchiolitis, and Visual Acuity independently, a respective 3 (2%), 10 (7%), and 0 records would have been missed. Abstrackr correctly predicted the relevance of 1 (33%) and 9 (90%) records missed by the second reviewer in the Antipsychotics and Bronchiolitis reviews, respectively. DistillerSR did not correctly predict the relevance of any of the records missed by the second reviewer in either review, thus providing no advantage over single-reviewer screening. RobotAnalyst correctly predicted the relevance of 4 (40%) records missed by the second reviewer in Bronchiolitis, but none of those missed in Antipsychotics.

#### Workload savings

**Fig. 5:**
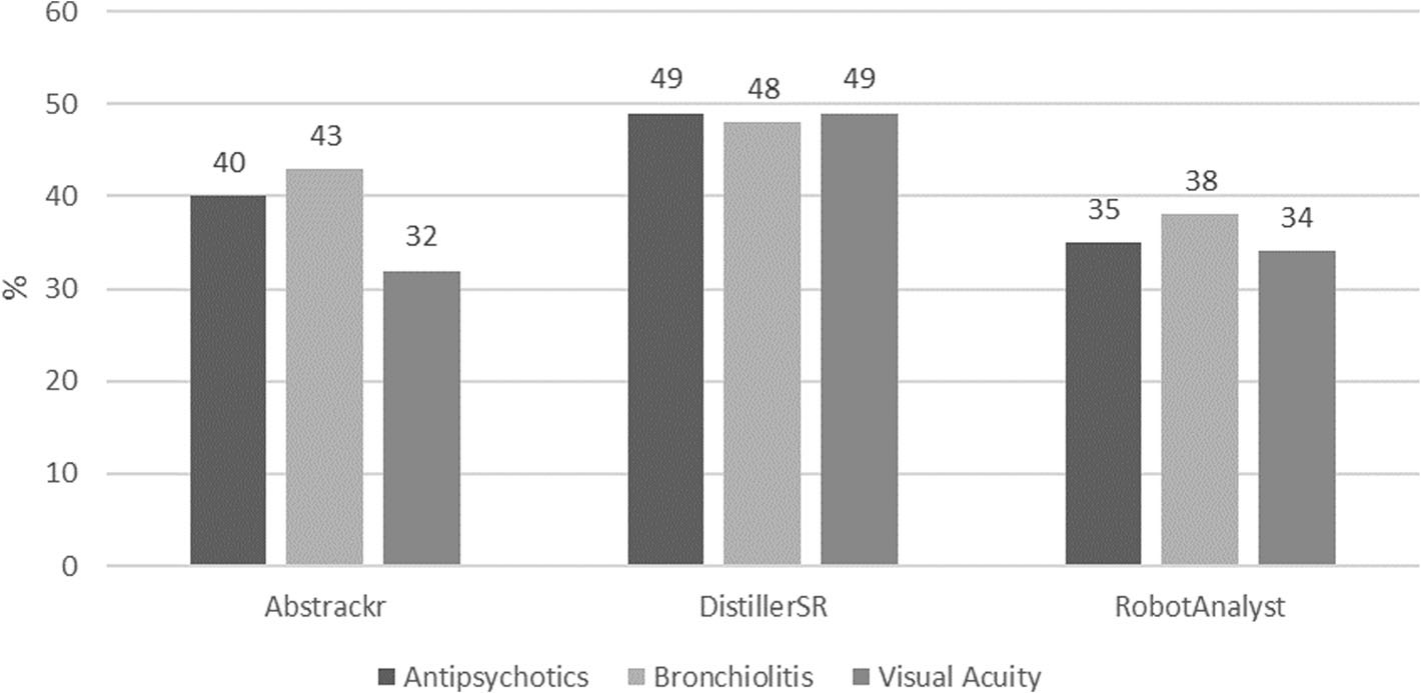
Workload savings (percent) by tool and systematic review, semi-automated simulation

The median (range) workload savings was 40 (32, 43)%, 49 (48, 49)%, and 35 (34, 38)% for Abstrackr, DistillerSR, and RobotAnalyst, respectively (Fig. 5).

#### Estimated time savings

**Fig. 6:**
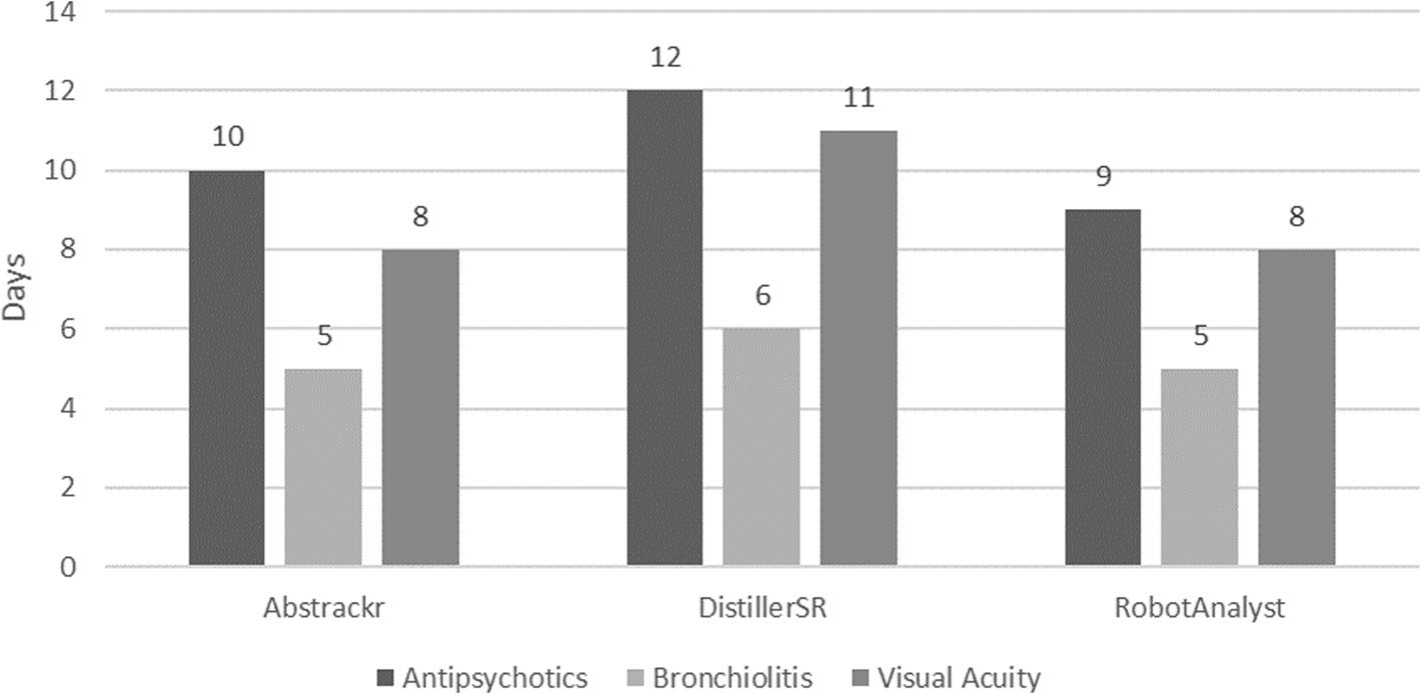
Estimated time savings (days) by tool and systematic review, semi-automated simulation

The median (range) time savings was 61 (42, 82), 92 (46, 100), and 64 (37, 71) hours for Abstrackr, DistillerSR, and RobotAnalyst, respectively (i.e., 8 (5, 10), 11 (6, 12), and 8 (5, 9) days) (Fig. 6).

### Post hoc analysis

Following our initial testing, we repeated the same procedures for a 500-record training set. We undertook the simulations for the larger training set only in Abstrackr, accounting for time and resource limitations. For the automated simulation, the median proportion missed increased from 28 to 41% for Antipsychotics and 5 to 9% for Bronchiolitis. There was no change in the proportion missed for Visual Acuity. The workload savings increased from 90 to 95% for Antipsychotics, 93 to 94% for Bronchiolitis, and 82 to 83% for Visual Acuity. The estimated time savings increased from 183 to 193 h for Antipsychotics, 91 to 92 h for Bronchiolitis, and 154 to 156 h for Visual Acuity.

For the semi-automated simulation, one additional record was missed for Antipsychotics; however, the proportion missed did not change. There was no change in the proportion missed for the other SRs. The workload savings increased from 40 to 45% for Antipsychotics, 43 to 44% for Bronchiolitis, and 32 to 33% for Visual Acuity. The estimated time savings increased from 82 to 92 h for Antipsychotics, 42 to 43 h for Bronchiolitis, and 61 to 62 h for Visual Acuity.

### User experiences

Eight research staff participated in the user experience testing (73% response rate). The median (interquartile range) SUS score was 79 (23), 64 (31), and 31 (8) for Abstrackr, DistillerSR, and RobotAnalyst, respectively (Table 3). Abstrackr fell in the usable, DistillerSR the marginal, and RobotAnalyst the unacceptable usability range [27]. Sixty-two percent of participants chose Abstrackr as their first choice and 38% as their second choice. Thirty-eight percent of participants chose DistillerSR as their first choice, 50% as their second choice, and 13% as their last choice. Thirteen percent of participants chose RobotAnalyst as their second choice and 88% as their last choice.

**Table 3:** System Usability Scale responses for each item, per tool^a^

Item	Abstrackr	DistillerSR	RobotAnalyst
I think that I would like to use the tool frequently	3.5 (1)	4 (0.5)	1 (1)
I found the tool to be unnecessarily complex	2 (1)	3.5 (1.25)	3 (0.5)
I thought the tool was easy to use	4 (1.25)	2.5 (2)	2 (1.5)
I think that I would need the support of a technical person to be able to use the tool	1 (1)	2.5 (1.25)	4 (1.25)
I found the various function in the tool were well integrated	4 (1.25)	3.5 (2.25)	3 (1.25)
I thought there was too much inconsistency in the tool	2 (0.25)	1 (1.25)	4 (1.25)
I would imagine that most people would learn to use the tool very quickly	4.5 (1)	3 (1.25)	3 (0.25)
I found the tool very cumbersome to use	2 (0.5)	3 (1.25)	5 (0)
I felt very confident using the tool	4 (1)	3.5 (1.25)	2 (2.25)
I needed to learn a lot of things before I could get going with the tool	2 (0.25)	3 (0.5)	2.5 (1)
Overall score (/100)	79 (23)	64 (31)	31 (8)

Likert-like scale: 1 = strongly disagree, 3 = neutral, and 5 = strongly agree. Values represent the median (interquartile range) of responses

The qualitative analysis revealed that usability was contingent on six interdependent properties: user friendliness, qualities of the user interface, features and functions, trustworthiness, ease and speed of obtaining the predictions, and practicality of the export files. Additional file 5 includes focused codes and participant quotes for each property.

Participants’ comments mirrored the quantitative findings. Most found Abstrackr to be easy to use. Although some described the user interface as “rudimentary,” participants generally appreciated that it was simple and lacked distractions. Many participants liked the customizability of review settings in Abstrackr, although some found it confusing and did not find the user guide to be helpful. Overall, Abstrackr was deemed relatively trustworthy, even if it was sometimes slow or crashed. Having to wait for the predictions was described as an “annoyance,” but not serious given the potential for time savings. There was little agreement as to whether Abstrackr’s export files were usable or practical.

Participants were divided with respect to DistillerSR’s user friendliness, with some finding it easy to use and others finding it unnecessarily complex. Although most liked the user interface, calling it “clean” and “bright,” others found it busy and overwhelming. Most participants felt that DistillerSR had too many features, making it feel sophisticated but overly complicated. Among the three tools, most participants found it to be the most reliable, referencing a professional look and feel, fast server speed, and few error messages. Many participants appreciated that the predictions were available in real time, but some could not figure out how to deploy them. DistillerSR’s output files were probably the most practical, but it often took a few attempts for participants to download them in the desired format.

RobotAnalyst was the least preferred. Most found it difficult to use due to a slow server speed, multiple pop-ups and error messages, and cumbersome screening process. The user interface, nevertheless, was described as “pretty” and participants liked the colors and layout. One participant appreciated that the relevance predictions appeared clearly on-screen, but otherwise the screening process was described as inefficient. Due to multiple glitches, most participants did not find the program to be trustworthy. As with DistillerSR, participants appreciated that the predictions were available in real time, but noted that applying them was slow. A positive comment about RobotAnalyst’s export files was that they were easy to download. Otherwise, participants consistently found the export to be impractical.

## Discussion

Supplementing a single reviewer’s decisions with Abstrackr’s predictions (semi-automated simulation) reduced the proportion missed compared with screening by the single reviewer, but performance varied by SR. Balanced with the potential for time savings, this approach could provide an acceptable alternative to dual independent screening in some SRs. By contrast, RobotAnalyst performed less well and DistillerSR provided no advantage over screening by a single reviewer. Differences between tools may reflect the relevance thresholds applied (we used standard settings) or differences in the ML algorithms. Replication on heterogeneous samples of reviews will inform when ML-assisted screening approaches may be worth the associated risk. Although the workload and time savings were superior when the tools were used to exclude irrelevant records (automated simulation), typically, far more studies were missed.

Empirical data show that learning increases quickly at the beginning of active learning and more slowly thereafter [29]. Thus, to obtain reliable predictions, large training sets can be required [14, 30]. It is unsurprising, then, that as a means to eliminate irrelevant records, the 200-record training produced unreliable predictions. Unfortunately, larger training sets may be impractical in real-world applications of ML tools. The 200-record training set was sufficient, in many cases, when paired with a single reviewer to capture ≥ 95% of relevant studies; however, this was not always an improvement over single reviewer screening. At present, the ideal training set size is unknown and likely review-specific [5]. In this study, Abstrackr’s predictions were most reliable for Bronchiolitis, which compared to Antipsychotics had fewer research questions and included only randomized trials. We speculate that ML may perform better for reviews with a single research question or those that include only randomized trials; however, our small sample precludes definitive conclusions.

Even if ML-supported screening approaches were ready to deploy, many review teams would remain hesitant pending widespread acceptance by methods groups, peer reviewers, grant panels, and journal editors [9]. Moving toward this ideal, there is a need for standard approaches to evaluating the performance and usability of the tools and reporting on these evaluations [7, 9, 31, 32]. Consistently conducted and reported evaluations will facilitate their replication across tools and SRs [31, 32], which will inform evidence-based guidance for their use [9]. The development of a set of outcome metrics, based on the consensus of end users (reviewers) and tool developers, may improve upon the value of future studies in this field. For example, the impact of missed studies on a SR’s conclusions is important to reviewers but less frequently considered by tool developers. Designing tools that allow reviewers to customize the level of risk (i.e., by setting their own relevance thresholds) may also contribute to garnering trust.

Another important contributor to the adoption of ML tools for screening will be their usability and fit with standard SR workflows [9]. The usability of the three tools varied considerably and relied upon multiple properties. Although usability will be of little concern once title and abstract screening is fully automated, the path toward that ideal begins with the acceptance and greater adoption of semi-automated approaches. Multiple experienced reviewers within our sample were unable to download the predictions from a number of the tools. Even when they were downloaded, the predictions were often in an impractical or unusable format. So long as reviewers cannot leverage the tools as intended, adoption is unrealistic. Greater attention to usability may improve the appeal of ML-assisted screening during early phases of adoption.

### Strengths and limitations

This is one of few studies to compare performance and user experiences across multiple ML tools for screening in SRs. Further, our study responds to a call from the International Collaboration for Automation of Systematic Reviews to trial and validate available tools [7] and addresses reported barriers to their adoption [9].

The training sets differed for each review across the tools. Although this could have affected the findings, in a previous evaluation, we found that Abstrackr’s predictions did not differ substantially across three trials [18]. In the absence of guidance for customizing the tools’ settings (e.g., deciding review-specific relevance thresholds), we used the standard settings in each tool to obtain predictions, which likely best approximated real-world use of the tools. We used a 200-record training set and a small sample of three SRs. The size of the training set can affect the resulting predictions. Our findings should not be generalized to other tools, SRs, or semi-automated screening approaches.

Time savings was estimated based on the reduced screening workload and a standard screening rate. This estimate did not account for time spent troubleshooting usability issues, nor for variability in the time spent screening records as reviewers progress through the screening task or for obviously excluded compared to records of uncertain relevance [29].

We did not investigate the impact of the missed studies on the results of the SRs. Future studies should plan for the time and resources to undertake these analyses in their protocols.

## Conclusions

Using Abstrackr’s predictions to complement the work of a single screener reduced the number of studies that were missed by up to 90%, although performance varied by SR. RobotAnalyst provided a lesser advantage, and Distiller provided no advantage over screening by a single reviewer. Considering workload and time savings, using Abstrackr to complement the work of a single screener may be acceptable in some cases; however, additional evaluations are needed before this approach could be recommended. Although using any tool to automatically exclude irrelevant records could save substantial amounts of time, the risk of missing larger numbers of relevant records is increased. The usability of the tools varied greatly. Further research is needed to inform how ML might be best applied to reduce screening workloads and to identify the types of screening tasks that are most suitable to semi-automation. Designing (or refining existing) tools based on reviewers’ preferences may improve their usability and enhance adoption.

## Supplementary information

**Additional file 1.** Screening exercise for the user experiences testing. Screening exercise instructions as presented to participants for the user experiences testing.

**Additional file 2.** User experiences survey. Details of the questions and response options on the user experiences survey.

**Additional file 3.** 2 × 2 tables and calculations for the performance metrics (example from the Antipsychotics review in Abstrackr). 2 × 2 tables and sample calculations for the proportion missed, workload savings, and estimated time savings for each simulation. This file shows an example from the Antipsychotics review in Abstrackr.

**Additional file 4.** 2 × 2 cross-tabulations for each review in each tool. 2 × 2 cross-tabulations for each review in each tool used to calculate the performance metrics.

**Additional file 5.** Focused codes and supporting quotes for the properties of each tool. Focused codes and supporting quotes for the themes that emerged from the qualitative analysis, for each tool.

## Data Availability

The datasets used and/or analyzed during the current study are available from the corresponding author on reasonable request.

## Abbreviations

ML: Machine learning
nRCT: Non-randomized controlled trial
PICOS: Population, Intervention, Comparator, Outcome, Study design
RCT: Randomized controlled trial
RIS: Research Information Systems
RSV: Respiratory syncytial virus
SR: Systematic review
