# Supplementary material for: Performance and usability of machine learning for screening in systematic reviews: a comparative evaluation of three tools
Source: Syst Rev. 2019 Nov 15;8:278. doi: 10.1186/s13643-019-1222-2 (PMC6857345; doi:10.1186/s13643-019-1222-2)
Supplement: Supplementary file 1 — Additional file 1. Screening exercise for the user experiences testing. Screening exercise instructions as presented to participants for the user experiences testing. [file 13643_2019_1222_MOESM1_ESM.docx]

Additional File 1. Screening Exercise for the User Experiences Testing

Screening Exercise

Before completing the survey, we ask that you undertake a brief screening exercise in each of the tools. The survey will ask you about your experiences using each tool, so keep note of anything that you like or dislike, or any difficulties that you encounter.

You will be screening records for a review on digital technologies for pain. The search found 2662 records. The RIS file, which contains all 2662 records, was attached to the invitation e-mail. The eligibility criteria for the review were also attached to the invitation e-mail.

Instructions for Each Tool

The goal of this exercise is to, in each tool: (a) create a project, (b) upload the studies to be screened, (c) screen a small training set, (d) download a record of the screening that you completed, and (e) download the predicted relevance of the remaining studies.

Feel free to investigate the various functions in each tool, and to use the “help” function if you are having trouble. Don’t worry if you cannot complete the task. We are interested in your experiences, both positive and negative.

The following includes instructions for each tool. Complete the exercise in each tool in the random order that was assigned to you.

Abstrackr

1. Navigate to the Abstrackr website: <http://abstrackr.cebm.brown.edu/account/login>
2. Register to create an account and log in.
3. Create a new project where one reviewer will screen the studies (“Single-screen” mode) and the studies will be presented in “most likely to be relevant” order.
4. Screen 100-200 studies.
5. Download a record of the decisions for the studies that you screened. Open the file to see what the output looks like. Consider whether this is practical format or not.
6. Check for the availability of predictions of the relevance of the remaining studies.
7. If no predictions are available, check back within 24 hours to see if they become available (the server updates overnight).
8. If predictions are available, download a record of the predicted relevance of the remaining studies. Open the file to see what the output looks like. Consider whether this is practical format or not.

DistillerSR

1. Navigate to the DistillerSR website: <https://v2dis-prod.evidencepartners.com/Login/Login.php>
2. Log in using the following username and password: [username] [password]
3. Contact Allison [email address] who will assign you to a project.
4. Upload the references from the RIS file to your project.
5. Screen 50 studies.
6. Download a record of the decisions for the studies that you screened from the “Datarama” as an Excel spreadsheet. Open the file to see what the output looks like. Consider whether this is practical format or not.
7. Check for the availability of predictions (“DistillerAI”). If they are not available, keep screening until they are ready.
8. Run DistillerAI using the “simple review” setting to generate predictions for the remaining studies.
9. Download a record of the predicted relevance of the remaining studies from “Datarama” as an Excel spreadsheet. Open the file to see what the output looks like. Consider whether this is practical format or not.

RobotAnalyst

1. Navigate to the RobotAnalyst website: http://nactem.ac.uk/RA/login.html
2. Log in using the following username and password: [username] [password]
3. Note: if you have trouble logging in, try reloading the page. If this does not work, try clearing your browser history.
4. Create a new collection where the default label is “undecided” and the studies are screened in random order.
5. Screen 50 studies.
6. Download a record of the decisions for the studies that you screened. Open the file to see what the output looks like. Consider whether this is practical format or not.
7. Update the predictions. If the predictions are not yet available, keep screening until they are ready.
8. Download a record of the predicted relevance of the remaining studies. Open the file to see what the output looks like. Consider whether this is a practical format or not.

You’re done! We would like to know what you thought about each of these tools. Please don’t forget to fill out the user experiences survey: [link to survey]
