# Supplementary material for: Performance and usability of machine learning for screening in systematic reviews: a comparative evaluation of three tools
Source: Syst Rev. 2019 Nov 15;8:278. doi: 10.1186/s13643-019-1222-2 (PMC6857345; doi:10.1186/s13643-019-1222-2)
Supplement: Supplementary file 2 — Additional file 2. User experiences survey. Details of the questions and response options on the user experiences survey. [file 13643_2019_1222_MOESM2_ESM.docx]

Additional File 2. User Experiences Survey

Thank you for taking part in this study to help us understand user experiences with machine learning tools for screening in systematic reviews (Pro00087862). As a reminder, your participation in this study is voluntary. Once you have completed the survey, it will be impossible to withdraw from the study.

By completing this survey, you agree that: you have read the information and recruitment letter and the study has been explained to you, you have been given the opportunity to ask questions and your questions have been answered, you have been told who to contact if you have further questions, and you agree to participate in the study as described in the recruitment and information letter. Completion of the survey will imply consent.

Thank you!

[Attachment: recruitment and information letter]

1. **Reflecting on your experiences screening in Abstrackr, to what extent do you agree with the following statements?** [scale of 1 (strongly disagree) to 5 (strongly agree)]
2. I think that I would like to use Abstrackr frequently.
3. I found Abstrackr to be unnecessarily complex.
4. I thought Abstrackr was easy to use.
5. I think that I would need the support of a technical person to be able to use Abstrackr.
6. I found the various functions in Abstrackr were well integrated.
7. I thought there was too much inconsistency in Abstrackr.
8. I would imagine that most people would learn to use Abstrackr very quickly.
9. I found Abstrackr very cumbersome to use.
10. I felt very confident using Abstrackr.
11. I needed to learn a lot of things before I could get going with Abstrackr.
12. **Please provide any positive or negative comments related to your experiences screening in Abstrackr.** [free-text responses]
13. **Reflecting on your experiences screening in DistillerSR, to what extent do you agree with the following statements?** [scale of 1 (strongly disagree) to 5 (strongly agree)]
14. I think that I would like to use DistillerSR frequently.
15. I found DistillerSR to be unnecessarily complex.
16. I thought DistillerSR was easy to use.
17. I think that I would need the support of a technical person to be able to use DistillerSR.
18. I found the various functions in DistillerSR were well integrated.
19. I thought there was too much inconsistency in DistillerSR.
20. I would imagine that most people would learn to use DistillerSR very quickly.
21. I found DistillerSR very cumbersome to use.
22. I felt very confident using DistillerSR.
23. I needed to learn a lot of things before I could get going with DistillerSR.
24. **Please provide any positive or negative comments related to your experiences screening in DistillerSR.** [free-text responses]
25. **Reflecting on your experiences screening in RobotAnalyst, to what extent do you agree with the following statements?** [scale of 1 (strongly disagree) to 5 (strongly agree)]
26. I think that I would like to use RobotAnalyst frequently.
27. I found RobotAnalyst to be unnecessarily complex.
28. I thought RobotAnalyst was easy to use.
29. I think that I would need the support of a technical person to be able to use RobotAnalyst.
30. I found the various functions in RobotAnalyst were well integrated.
31. I thought there was too much inconsistency in RobotAnalyst.
32. I would imagine that most people would learn to use RobotAnalyst very quickly.
33. I found RobotAnalyst very cumbersome to use.
34. I felt very confident using RobotAnalyst.
35. I needed to learn a lot of things before I could get going with RobotAnalyst.
36. **Please provide any positive or negative comments related to your experiences screening in RobotAnalyst.** [free-text responses]
37. **Considering your experiences with the three tools, which would you prefer to use for screening in a systematic review?**
38. Abstrackr [first choice, second choice, or third choice]
39. DistillerSR [first choice, second choice, or third choice]
40. RobotAnalyst [first choice, second choice, or third choice]
41. **Which features of any of the tools support their usability and appeal? Please explain.** [free-text responses]
42. **Which features of any of the tools hinder their usability and appeal? Please explain.** [free-text responses]
43. **If you any additional comments related to your experiences with the three tools, please include them here.** [free-text responses]
