# Supplementary material for: Performance and usability of machine learning for screening in systematic reviews: a comparative evaluation of three tools
Source: Syst Rev. 2019 Nov 15;8:278. doi: 10.1186/s13643-019-1222-2 (PMC6857345; doi:10.1186/s13643-019-1222-2)
Supplement: Supplementary file 3 — Additional file 3. 2 × 2 tables and calculations for the performance metrics (example from the Antipsychotics review in Abstrackr). 2 × 2 tables and sample calculations for the proportion missed, workload savings, and estimated time savings for each simulation. This file shows an example from the Antipsychotics review in Abstrackr. [file 13643_2019_1222_MOESM3_ESM.docx]

Additional File 3. 2x2 Tables and Calculations for the Performance Metrics (Example from the Antipsychotics Review in Abstrackr)

2x2 Cross-tabulations

Automated Simulation:

|  | Excluded from final report | Included in final report | Row total |
| --- | --- | --- | --- |
| Excluded by Simulation | 11450 | 36 | 11486 |
| Included by Simulation | 579 | 91 | 670 |
| Column total | 12029 | 127 | 12156 |

Semi-automated Simulation:

|  | Excluded from final report | Included in final report | Row total |
| --- | --- | --- | --- |
| Excluded by Simulation | 11101 | 2 | 11103 |
| Included by Simulation | 928 | 125 | 1053 |
| Column total | 12029 | 127 | 12156 |

Predictions were available after screening 200 records. Abstrackr predicted that 2117 of the remaining records were relevant and 9839 were irrelevant.

Sample Calculations

Proportion Missed (i.e., error)

Of the studies included in the final report, the proportion that would have been excluded at title and abstract screening for each simulation.

Automated Simulation: proportion missed = 36 / 127 = 0.28 or 28%

Semi-automated Simulation: proportion missed = 2 / 127 = 0.016 or 1.6%

Workload savings (i.e., absolute screening reduction)

Of the number of records that would need to be screened at the title and abstract stage (assuming dual independent screening), the proportion that would not need to be screened manually.

Automated Simulation: workload savings = (9839 + 12156) / (12156 x 2) = 0.90 or 90%

Semi-automated Simulation: workload savings = 9839 / (12156 x 2) = 0.40 or 40%

Time Savings

The time saved by not screening records manually, assuming a screening rate of 0.5 minutes per record and an 8-hour work day.

Automated Simulation: time savings = [(9839 +12156) x 0.5 min/record] x 1 hour/60 min x 1 day/8 hours = 23 days

Semi-automated Simulation: time savings = (9839 x 0.5 min/record) x 1 hour/60 min x 1 day/8 hours = 10 days
