# Supplementary material for: Performance and usability of machine learning for screening in systematic reviews: a comparative evaluation of three tools
Source: Syst Rev. 2019 Nov 15;8:278. doi: 10.1186/s13643-019-1222-2 (PMC6857345; doi:10.1186/s13643-019-1222-2)
Supplement: Supplementary file 4 — Additional file 4. 2 × 2 cross-tabulations for each review in each tool. 2 × 2 cross-tabulations for each review in each tool used to calculate the performance metrics. [file 13643_2019_1222_MOESM4_ESM.docx]

Additional File 4. 2 x 2 Cross-tabulations for Each Review in Each Tool

2x2 Tables for the Automated Simulation

Antipsychotics, Abstrackr

|  | Excluded from final report | Included in final report | Row total |
| --- | --- | --- | --- |
| Excluded by Simulation | 11450 | 36 | 11486 |
| Included by Simulation | 579 | 91 | 670 |
| Column total | 12029 | 127 | 12156 |

Antipsychotics, DistillerSR

|  | Excluded from final report | Included in final report | Row total |
| --- | --- | --- | --- |
| Excluded by Simulation | 12011 | 123 | 12134 |
| Included by Simulation | 18 | 4 | 22 |
| Column total | 12029 | 127 | 12156 |

Antipsychotics, RobotAnalyst

|  | Excluded from final report | Included in final report | Row total |
| --- | --- | --- | --- |
| Excluded by Simulation | 8558 | 89 | 8647 |
| Included by Simulation | 3471 | 38 | 3509 |
| Column total | 12029 | 127 | 12156 |

Bronchiolitis, Abstrackr

|  | Excluded from final report | Included in final report | Row total |
| --- | --- | --- | --- |
| Excluded by Simulation | 5559 | 7 | 5566 |
| Included by Simulation | 165 | 130 | 295 |
| Column total | 5724 | 137 | 5861 |

Bronchiolitis, DistillerSR

|  | Excluded from final report | Included in final report | Row total |
| --- | --- | --- | --- |
| Excluded by Simulation | 5685 | 131 | 5816 |
| Included by Simulation | 39 | 6 | 45 |
| Column total | 5724 | 137 | 5861 |

Bronchiolitis, RobotAnalyst

|  | Excluded from final report | Included in final report | Row total |
| --- | --- | --- | --- |
| Excluded by Simulation | 5548 | 31 | 5579 |
| Included by Simulation | 176 | 106 | 282 |
| Column total | 5724 | 137 | 5861 |

Visual Acuity, Abstrackr

|  | Excluded from final report | Included in final report | Row total |
| --- | --- | --- | --- |
| Excluded by Simulation | 11109 | 0 | 11109 |
| Included by Simulation | 119 | 1 | 120 |
| Column total | 11228 | 1 | 11229 |

Visual Acuity, DistillerSR

|  | Excluded from final report | Included in final report | Row total |
| --- | --- | --- | --- |
| Excluded by Simulation | 11226 | 1 | 11227 |
| Included by Simulation | 2 | 0 | 2 |
| Column total | 11228 | 1 | 11229 |

Visual Acuity, RobotAnalyst

|  | Excluded from final report | Included in final report | Row total |
| --- | --- | --- | --- |
| Excluded by Simulation | 11148 | 1 | 11149 |
| Included by Simulation | 80 | 0 | 80 |
| Column total | 11228 | 1 | 11229 |

2x2 Tables for Semi-automated Simulation

Antipsychotics, Abstrackr

|  | Excluded from final report | Included in final report | Row total |
| --- | --- | --- | --- |
| Excluded by Simulation | 11101 | 2 | 11103 |
| Included by Simulation | 928 | 125 | 1053 |
| Column total | 12029 | 127 | 12156 |

Antipsychotics, DistillerSR

|  | Excluded from final report | Included in final report | Row total |
| --- | --- | --- | --- |
| Excluded by Simulation | 11165 | 2 | 11167 |
| Included by Simulation | 864 | 125 | 989 |
| Column total | 12029 | 127 | 12156 |

Antipsychotics, RobotAnalyst

|  | Excluded from final report | Included in final report | Row total |
| --- | --- | --- | --- |
| Excluded by Simulation | 7980 | 3 | 7983 |
| Included by Simulation | 4049 | 124 | 4173 |
| Column total | 12029 | 127 | 12156 |

Bronchiolitis, Abstrackr

|  | Excluded from final report | Included in final report | Row total |
| --- | --- | --- | --- |
| Excluded by Simulation | 5357 | 1 | 5358 |
| Included by Simulation | 367 | 136 | 503 |
| Column total | 5724 | 137 | 5861 |

Bronchiolitis, DistillerSR

|  | Excluded from final report | Included in final report | Row total |
| --- | --- | --- | --- |
| Excluded by Simulation | 5394 | 10 | 5404 |
| Included by Simulation | 330 | 127 | 457 |
| Column total | 5724 | 137 | 5861 |

Bronchiolitis, RobotAnalyst

|  | Excluded from final report | Included in final report | Row total |
| --- | --- | --- | --- |
| Excluded by Simulation | 5364 | 6 | 5370 |
| Included by Simulation | 360 | 131 | 491 |
| Column total | 5724 | 137 | 5861 |

Visual Acuity, Abstrackr

|  | Excluded from final report | Included in final report | Row total |
| --- | --- | --- | --- |
| Excluded by Simulation | 11075 | 0 | 11075 |
| Included by Simulation | 153 | 1 | 154 |
| Column total | 11228 | 1 | 11229 |

Visual Acuity, DistillerSR

|  | Excluded from final report | Included in final report | Row total |
| --- | --- | --- | --- |
| Excluded by Simulation | 11074 | 0 | 11074 |
| Included by Simulation | 154 | 1 | 155 |
| Column total | 11228 | 1 | 11229 |

Visual Acuity, RobotAnalyst

|  | Excluded from final report | Included in final report | Row total |
| --- | --- | --- | --- |
| Excluded by Simulation | 11042 | 0 | 11042 |
| Included by Simulation | 186 | 1 | 187 |
| Column total | 11228 | 1 | 11229 |
