# Supplementary material for: Performance and usability of machine learning for screening in systematic reviews: a comparative evaluation of three tools
Source: Syst Rev. 2019 Nov 15;8:278. doi: 10.1186/s13643-019-1222-2 (PMC6857345; doi:10.1186/s13643-019-1222-2)
Supplement: Supplementary file 5 — Additional file 5. Focused codes and supporting quotes for the properties of each tool. Focused codes and supporting quotes for the themes that emerged from the qualitative analysis, for each tool. [file 13643_2019_1222_MOESM5_ESM.docx]

Additional File 5. Focused Codes and Supporting Quotes for the Properties of Each Tool

Comments Related to Abstrackr

| Properties and focused codes | Supporting quotes |
| --- | --- |
| **User friendliness**  *Positives:* user friendly/easy to screen records; easy to navigate; easy to make a new review; relatively simple program; makes screening more enjoyable  *Negatives:* difficult to figure out how to upload records; changing the review settings is arduous; not very intuitive; not the most user friendly; interface could be improved to streamline processes for project management | “The user interface is relatively appealing, but not distracting. This is a relatively simple program, and when it works, it makes screening a bit more enjoyable.”  “Very rudimentary graphics, display and options, but this also made Abstrackr very easy to use; probably the easiest to use as not many functionalities and very easy to find […]”  “Easy to use for screening but not very intuitive when trying to export records or go back to main page […]”  “Instructions said to create project where studies would be presented in "most likely to be relevant" order - I couldn't figure out how to select that option.” |
| **Qualities of the user interface**  *Positives:* appealing user interface; not distracting; nice look and layout  *Negatives:* not the most pretty looking; very rudimentary graphics and display | “I liked the look; lay-out for screening; easy to use and advanced to next record easily and quickly.”  “I wanted to say also that this tool is not the most pretty looking; user friendly, but does at least appear trustworthy, so I would use it […]” |
| **Features and functions**  *Positives:* can add notes and tags to the records; can select single or dual screen mode; can change the order of the records; can change decision in the case of mistakes  *Negatives:* user guide is not that helpful; unclear how to change the order of the records; record IDs not shown; unclear how or if the most likely to be relevant prioritization works; unclear if decisions file will include tags; unclear how the tagging works | “For first timers, it is difficult to figure out how to upload the records from EndNote into Abstrackr, and the user help guide was not very helpful.”  “I do like that you can tag studies (e.g., SRs) but not sure how it all works or if the decisions file actually displays these.”  “I also like that you can change your decisions if you make a mistake, but the process to do so is a bit cumbersome (have to go through a few pages).” |
| **Trustworthiness**  *Positives:* appears trustworthy; would use again; advanced to next record easily and quickly  *Negatives:* sometimes slow or crashes; unclear error messages; unpredictable user interface; server seems slow; delays in uploading records | “I wanted to say also that this tool is not the most pretty looking; user friendly, but does at least appear trustworthy, so I would use it again.”  “When uploading the records, the program is sometimes very slow or can crash. You get a bright orange screen with little indication as to what you may have done wrong. Spontaneously (or so it seems), the program will start working again.” |
| **Ease and speed of obtaining the predictions**  *Positives:* waiting for the predictions is not a deal breaker/not a big issue given the time savings  *Negatives:* slow to develop predictions; requires a larger training set than other programs; predictions cannot be updated manually; must wait overnight (or hours) for predictions, which is long compared to other programs; no way to know how many records will need to be screened before you get predictions | “One last thing is that it is a bit annoying to have to wait a day for the predictions to appear. This is especially the case since there is no way to know how many records will need to be screened before you get predictions.”  “Compared to the other programs, it seems like Abstrackr requires a larger training set before providing predictions. Also, the predictions cannot be updated manually by the user; instead, one must wait overnight for the predictions to be produced. This is a little bit inconvenient, but probably not a deal breaker for me.”  “Waiting time on predictions is a bit lengthy compared to other machine learning programs but considering the time saved in screening overall it isn't a big issue.” |
| **Practicality of the export file(s)**  *Positives:* consensus column; contains information that is helpful when reviewing the screening decisions (e.g., title, authors); seems usable and practical; could easily import into a different format or program  *Negatives:* year published is missing; does not always include the original reference IDs; format is not user friendly; would need a lot of work before they would be usable; hard to use/might be tedious; use might be prone to error; unclear why -1 and 1 labels are used | “Output format seems practical as it contains the authors, titles and abstracts which will be helpful in reviewing screening decisions”  “The format for downloading the predictions and screening record is not very user friendly. Sometimes, the file does not include the original reference IDs […]”  “Downloaded record of decisions for screened studies - into excel using CSV format; format seemed usable and could likely import this easily into other formats; programs.”  “[…] quite a bit of work is required to reformat the files before they would be usable for a reviewer.”  “I did not find the coding in the Excel spreadsheet explained anywhere (0, 1, -1); found the export to be practical” |

Comments Related to DistillerSR

| Properties and focused codes | Supporting quotes |
| --- | --- |
| **User friendliness**  *Positives:* user friendly; easy to use; easy to navigate; could find right options after spending time looking at menus; easy to track progress (record IDs follow flow of records)  *Negatives:* additional features make screening cumbersome; may require training to use it more efficiently; requires more skill to set up; unnecessarily complex; sometimes difficult to navigate; initially did not know where to find needed functions; had to watch a tutorial to set up the screening process | “Once everything is set up the screening is very easy and the process quite user-friendly”  “Distiller has a very appealing user interface, and once the project is set up, it is pretty easy to use. That said, it requires much more skill to actually set up a project in Distiller compared to the other programs. Since Distiller has so many more functions […] for screening it is more cumbersome than the other two programs.”  “The format of screening is nice, clear and consistent (e.g., abstract is organized with white space). During screening, the order of references followed the order of screening so it was easy to track progress.” |
| **Qualities of the user interface**  *Positives:* very appealing user interface; clean and bright; easy interface for screening; clear; consistent; abstract is organized with white space; very nice to look at  *Negatives:* a lot “going on”/a lot of information on each screen and on drop-down menus; a bit overwhelming at first | “My favourite part about this program is probably its clean and bright user interface […]”  “[…] the interface is very nice to look at.”  “[…] a lot of information on each screen and dropdown menu so initially did not know where to find the needed functions”  “[…] I liked the interface and colours [..]” |
| **Features and functions**  *Positives:* tutorial videos are quite helpful; ”drop files” function is a great feature; help function is very useful  *Negatives:* too many features; not easy to go back and fix mistakes; requires two clicks to make a decision; unclear why there are two “submit form” buttons; tutorial videos went through things too slowly; having choices listed consecutively (i.e., vertically compared to horizontally) could lead to errors | “Distiller seemed like a very sophisticated tool, with lots of options etc. to choose from, which made it a bit overwhelming at first.”  “[…] the software has too many features that makes it look like unnecessarily complex.”  “[…] and I liked the little ? symbols which provided tips and explanations.”  “Distiller seemed complex, there was a lot "going on" and sometimes I found it hard to navigate through the different pages, project data sheets, etc”  “The "drop files" function for uploading records was a great feature.” |
| **Trustworthiness**  *Positives:* more professional looking than Abstrackr; server is very fast and responsive; reliable/trustworthy; advances well | “[…] it was more professional looking than Abstrackr.”  “My favourite part about this program is probably its clean and bright user interface, quick server, and reliability.”  “Of the tools I think that this one is the one that I would trust the most with my records. It seemed to have a professional backing to it.” |
| **Ease and speed of obtaining the predictions**  *Positives:* predictions become available quickly; predictions can be applied in a matter of seconds; running DistillerAI was easy; faster than Abstrackr  *Negatives:* seems ahead of its time; unclear if predictions can be removed from the review; couldn’t figure out how to get predictions; took me forever to find Distiller AI; need a tutorial on best settings to use for DistillerAI; unclear how to know best threshold for setting the predictions | “Predictions are much faster than Abstrackr, and are available for all studies in less than ten minutes.”  “[…] AI feature became available after 106 screens”  “Running the AI was relatively easy, though if I were actually to go through with it I feel like I could use a tutorial on the best settings to use. They seem a bit ahead of the times - you can choose the 'accuracy' of the prediction but how do I know what would work best?”  “I don't know if you can 'undo' the AI once it's done but that would be nice.” |
| **Practicality of the export file(s)**  *Positives:* great variety of output formats; almost instantaneous output to Excel; download output is very practical; download output is very useful; highly user friendly; would not require much formatting to be usable; not overly complicated  *Negatives:* initial output didn’t have the information I needed/needed to select correct display options; unsure what “coding terms” meant; lacking author, title, abstract; couldn’t figure out how to export the predictions; unnecessarily complicated; missing important information that would make it more usable | “I liked how Distiller had a great variety of output formats for the screening decisions and predictions […].”  “Output to Excel file was almost instantaneous”  “[…] the Excel file that can be exported is highly user friendly and would not require much formatting to be in a usable format for a review.”  “Downloading the records was quite easy and the output looks practical to use - basically the REF IDs and the decisions in 2 columns.”  “Output is less helpful for reviewing decisions as the author, title and abstract are not included when exported to Excel.”  “I seem to have generated predictions but couldn't figure out how they would be exported.” |

Comments Related to RobotAnalyst

| Properties and focused codes | Supporting quote(s) |
| --- | --- |
| **User friendliness**  *Positives:* looks easy to navigate; it was intuitive; it was easy to use; screening form is easy to use and understand; uploading records is simple  *Negatives:* meaning of pop-ups and error messages was unclear; pop-ups and error messages were very distracting; screening decisions would disappear and had to re-screen; very difficult to work with; logging in was difficult; screening is cumbersome; uploading the records was a hassle due to slow server speed; did not like having to scroll down to see records; difficult to attempt to track records for which decisions have been recorded; cumbersome | “After screening records, they change colour; however, then they would change back and it would appear like they had not yet been screened. I was not really sure what to make of it. Uploading the records is a hassle, because the server is so slow.”  “Firstly, the web page is extremely slow, even when performing minor tasks like simply logging in. Uploading the records is simple but ridiculously slow considering it was a relatively small set of records. The fact that you have to actually click on each record to see the abstract is very cumbersome and does not make any sense if the purpose of the program is to facilitate screening. Once you can see the whole record, the drop-down menu to choose include; exclude is cumbersome - would be better to have a radio box (not sure what that's called). The program also took several seconds to register a decision. Then, out of nowhere, it would randomly lose all the previous decisions on the page.” |
| **Qualities of the user interface**  *Positives:* the user interface is okay; landing page looks very nice; liked the use of colours and buttons; very pretty; I liked the layout  *Negatives:* a bit busy | “Slow to load but very pretty! I liked the use of colours and buttons […]”  “The user interface is okay, but a little busy.” |
| **Features and functions**  *Positives:* predictions were available on-screen during the screening process  *Negatives:* had to open each record to read the abstract; constant pop-up to update the predictions is annoying; does not automatically advance to the next record; lack of logical numbers for records; drop down menu to choose include or exclude is cumbersome; impossible to tell status of upload | “A major downfall of this program is that you need to click on the title to see the abstract, unlike other tools where the abstract and title automatically appear on-screen. This is especially cumbersome given the slow server speed.”  “Once the predictions are ready, there is a constant pop-up every few records reminding you to update them. It is a little annoying.”  “[…] lack of logical order of record numbers makes it additionally difficult to attempt to track records for which decisions had been recorded […]” |
| **Trustworthiness**  *Negatives:* unable to complete the screening unsure what to make of the screening process; seemed glitchy; unreliable; sometimes had to click twice for the screening decisions; seems untrustworthy; program was extremely slow; took several seconds to register a decision; randomly loses all previous decisions on a page; colours would disappear and unclear if decisions had been lost; could not find a way to tag records; steady stream of error messages; could not trust if decisions were being registered; logging in took multiple attempts; sometimes the wrong record opened; program said files were uploaded but they were not; error stops all screening and asks you to contact admin, then resolves itself; takes longer to screen studies due to time waiting for abstracts to load; slowest program of the three; worry that on-screen predictions could bias the screening process; required a few tries to download the predictions | “Logging in was sometimes difficult; the program would seem to shut down and I would need to reset my browser history to get it working again. Once logged in, the server was extremely slow, and there were multiple pop-ups and error messages, none of which I could quite figure out the meaning of, that were highly distracting.”  “I was getting several error messages while screening, and was unable to complete the screens”  “I sort of like that the predictions appear on-screen alongside the records as you are screening; however, I sort of worry whether this would bias the human's screening decisions.”  “I would not use this program, mostly because it is cumbersome and does not seem trustworthy at all.”  “I would never use this program for a systematic review. It is completely unreliable and there is no practical way to download the screening decisions or predictions.” |
| **Ease and speed of obtaining the predictions**  *Positives:* predictions were available quickly  *Negatives:* applying the predictions was slow | “Seemed to be quick to apply predictions.”  “Applying the predictions was slow, which was not too surprising by that point.” |
| **Practicality of the export file(s)**  *Positives:* Exporting the decisions was not hard  *Negatives:* download does not contain the screening decisions and predictions; output download is not practical; predictions had to be added to the download file manually; export file is not usable; report was impossible to understand; record of decisions was poorly organized; difficult to decipher; text download file is useless; did not know what to do with the export file | “Exporting the decisions was not hard, but required a few tries since the first time the program seemed to be thinking for awhile but nothing downloaded. The next time it worked.”  “When it comes to downloading the predictions I could not find any practical way to do so, such that they would be in a format that could actually be used. The download is actually a .txt file, which can be converted to .ris and opened in EndNote. That said, there is nowhere in this EndNote file where the screening decisions can be found. These have to be added in manually.”  “[…] record of decisions in notepad poorly organized and difficult to decipher.” |
